# Self-Expandable Metal Stenting of Refractory Upper Gut Corrosive Strictures: A New Role for Endoscopy?

**DOI:** 10.1155/2011/346413

**Published:** 2011-07-17

**Authors:** Raffaele Manta, Rita Conigliaro, Helga Bertani, Mauro Manno, Ahmed Soliman, Paolo Fedeli, Gabrio Bassotti

**Affiliations:** ^1^Gastroenterology and Endoscopy Unit, S. Agostino Estense Civil Hospital, Baggiovara Modena, Italy; ^2^Gastroenterology Unit, Santo Spirito Hospital, Rome, Italy; ^3^Gastroenterology & Hepatology Section, Department of Clinical & Experimental Medicine, Perugia Medical University School, Italy

## Abstract

Caustic strictures of the gastrointestinal tract are often difficult to treat, since relapses are frequent after medical or endoscopic treatment. Thus, novel approaches are needed. We report here our experience with self-expandable metallic stents (SEMS) as a new endoscopic approach in three patients with corrosive strictures of the upper gastrointestinal tract.

## 1. Introduction

Caustic injury to the upper gastrointestinal (GI) tract represents a significant medical and social problem [1]. In pediatric patients, caustic ingestion is usually accidental [2], whereas in adults, it is more often intentional, associated with suicide attempts, and usually involves strong acids [3, 4].

Although mortality is not high, there is a highly related morbidity rate: caustic ingestion may thus cause perforations and necrosis in the acute phase, while the late complications include stricture formation, especially in the esophagus and the antrum, and the development of esophageal and, rarely, gastric carcinoma [5, 6].

Endoscopic management of caustic strictures of the esophagus and the gastric antrum usually relies on dilatation procedures [7–9], although alternative procedures may be used as a first therapeutic choice or when dilatation is not effective [10, 11].

In recent years, the use of endoscopic stenting for nonneoplastic stenoses has been implemented, in both pediatric and adult subjects [12–15].

We report three cases of corrosive injury of the upper GI tract treated by self-expandable metal stents (SEMS) positioning.

## 2. Case Series

### 2.1. Patient 1

**Figure 1:**
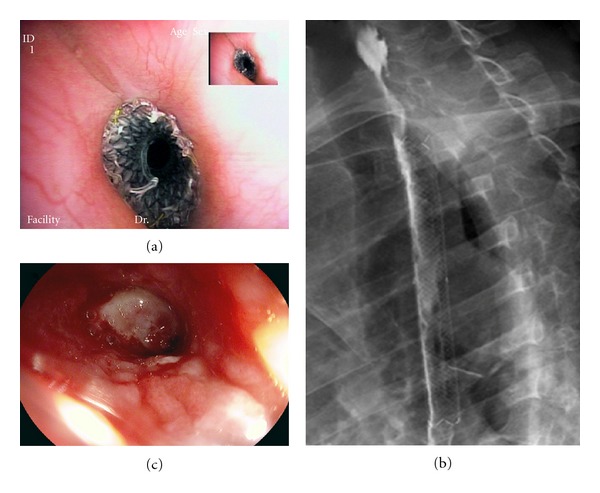
(a) Conio stent placed across the cervical stenosis. (b) Radiologic control of stent positioning by water soluble contrast medium. (c) Cervical esophageal lumen after stent removal: beyond the stenotic tract a decubitus ulcer is seen.

A 50-year old woman with bipolar manic-depressive syndrome was referred to our Endoscopy Unit after voluntary ingestion of an unidentified volume and concentration of muriatic acid. On admission, her main complaint was chest pain; physical examination was normal. Chest and abdomen X-ray films did not reveal any abnormalities, and water soluble CT scan did not show perforation. Upper endoscopy performed in the first 24 hours after ingestion showed multiple ulcers with extensive necrosis of esophagus and stomach, while the duodenum was edematous with some erosions and superficial ulcerations (stage 3B and 2A, resp., according to the classification of Zargar and colleagues [16], Table 1). A central venous catheter was inserted for nutritional support, and the patient was treated with antibiotics and omeprazole, 40 mg bid, i.v. The patient was discharged one week later on oral omeprazole therapy. After a month, she was admitted again due to vomiting. Upper GI endoscopy, performed by a pediatric instrument (Pentax EG-2930K), revealed two substenotic tracts of the esophagus 15 and 35 cm from the incisors. Fragility of gastric mucosa and a 7 cm antropyloric stricture were also observed. An upper GI series revealed a decreased calibre of the distal esophagus, the distal part of the antrum, and pylorus. Pneumatic balloon dilation (CRE Boston Scientific diameter 12–15 mm, at 6 ATM) of the antropyloric stenosis was carried out and repeated (balloon diameter 18–20 mm, 4–6 ATM) after a month for recurrence of a 5 cm stenosis. At one-month followup, the stenosis was still present and was retreated by balloon dilatation (diameter 12–15 mm at 6 ATM) followed by SEMS positioning (Taewoong pyloric covered metallic stent, 18 × 60 mm). After three months, when planned SEMS extraction, the patient complained of dysphagia. Endoscopy showed a cervical stricture and a double stricture of the medium esophagus (22 and 30 cm from incisors, resp.), the latter successfully treated in one session by Savary dilators (9 to 16 mm) under fluoroscopic guidance, followed by intralesional corticosteroids injection (triamcinolone acetonide 40 mg/10 mL). The cervical stricture was treated in five endoscopic sessions by balloon dilation (diameter up to 18 mm, 4, 5 ATM) and thereafter by SEMS (Taewoong Conio-type stent: 16 mm, 12 cm) placement because of the stricture recurrence (Figures 1(a) and 1(b)). The stent dislodged distally after 2 days and was removed endoscopically on the 3rd day (Figure 1(c)). Restenosis did not occur after stent dislocation. Three months later the pyloric metallic stent was removed after freeing it with argon plasma coagulation (APC). At 24-month followup no stricture recurrence was observed. 

### 2.2. Patient 2

A 56-year old schizophrenic woman was referred to our Endoscopy Unit with a diagnosis of pyloric stenosis due to voluntary ingestion of about 500 mL of caustic substance (benzalconium chloride and muriatic acid in an unknown concentration). *Esophagogram with water soluble contrast did not show perforation of the viscus.* Upper GI endoscopy showed hyperemia and edema of the prepyloric and pyloric mucosa with narrowed but passable pylorus by a standard endoscope. The mucosal injury was classified as grade 1 according to the above classification. Total parenteral nutrition (TPN) was started together with i.v. omeprazole, 40 mg bid. At one-month followup, endoscopy showed pyloric stricture. An upper GI series showed a 4 cm long prepyloric stenosis. Pneumatic dilatation using CRE balloon (Boston Scientific, diameter 12–15 at 6 ATM) was performed in three consecutive sessions, and a covered SEMS (Taewoong Pyloric 18 mm, 6 cm) was placed under fluoroscopic guidance for stricture recurrence. The patient was discharged after two days, and omeprazole, 20 mg bid, was prescribed. Three months later the SEMS was removed after cutting and freeing with a clamp tissue overgrowth at the edges of the metallic mesh. Subsequent endoscopy and upper GI series revealed resolution of the stenosis. After three months, endoscopic followup showed a substenosis of the pyloric tract that was retreated by balloon dilation (up to 18 mm diameter at 4.5 ATM). The patient was asymptomatic after 6 months of followup.

### 2.3. Patient 3

A 38-year old man was admitted at our Endoscopy Unit after voluntary ingestion of an unidentified volume and concentration of muriatic acid. *A CT scan with water soluble contrast did not show esophageal perforation.* Upper GI endoscopy showed hyperemia, edema, and friability associated to erosions of the cervical esophagus and extensive necrosis of the stomach. The duodenal bulb was very edematous with multiple erosions and superficial ulcerations (stages 2A and 3B, resp., according to the above classification). TPN was started together with i.v. omeprazole, 40 mg bid, and antibiotic therapy. After 20 days, GI endoscopy showed a double stenosis of cervical esophagus and a pyloric stricture impassable with a paediatric instrument. An upper GI series showed a 5.5 cm long prepyloric stenosis. Mechanic dilatation (Savary) up to 16 mm diameter followed by pneumatic dilatation with CRE balloon (Boston Scientific, 12–15 mm diameter at 4 and 6 ATM) was performed, respectively, for esophageal and pyloric strictures in two consecutive sessions. Thereafter, a covered SEMS (Taewoong Pyloric 20 mm, 8 cm length) was positioned in the antrum under fluoroscopic guidance, while the esophageal stenosis was resolved. The patient was discharged after one week with omeprazole therapy, 20 mg bid. Two months later an endoscopic control showed that the SEMS was almost free in the gastric cavity, and it was extracted. Subsequently, an upper GI series revealed resolution of the stenosis. After three months endoscopy showed narrowing of the pyloric channel, well passable with a standard endoscope (Pentax G-3030K). The patient was asymptomatic after 6 months of followup.

## 3. Discussion

Corrosive agents are acid or alkaline compounds, with a pH less than 2 or greater than 12 [6]. Alkali ingestion causes more commonly esophageal injuries, whereas the damage caused by acids is maximal in the stomach, and the esophagus is minimally affected [6, 17–19]. Both alkali and acids penetrate tissues extremely rapidly and cause full thickness damage to the gastrointestinal wall [6, 20, 21]. The extent and severity of caustic injury depends on the pH of the corrosive agent, its quantity and concentration, the physical state of the caustic substance, the duration of exposure, and the subsequent secondary infection [22]. 

Mucosal damage can occur up to 7 days after initial injury, and then bacterial invasion, inflammatory response, and development of granulation tissue ensue [23]. Scar retraction begins in the third week and continues for several months thereafter, leading to stricture and shortening of the damaged segment [24, 25].

The initial assessment usually starts with a plain lateral neck and chest radiographs, to exclude perforation; in the absence of perforation, endoscopy is the diagnostic procedure of choice [19, 26]. Endoscopy should be performed not prior than 6 hours (preferably within 12 hours) after caustic ingestion, due to the high risk of iatrogenic damage, and avoided 5 to 15 days after the ingestion, due to wound softening during this period [16, 17, 23]. Identification of the initial damage severity is of paramount importance for management and prognosis, since it allows to distinguish patients with no evidence of GI injury, that can be discharged, from patients with severe injuries [17]. 

After ingestion of corrosive substances, 38% to 45% of patients develop esophageal strictures; gastric outlet obstruction is relatively uncommon, and it is usually found in association with esophageal strictures (20% of cases) [17, 27]. 

Concerning management, although many treatment modalities have been suggested to prevent the development of such complications, none has yielded univocal success [28–31]. 

Periodic endoscopic dilation with bougies or balloons in alternative to surgery is the traditional treatment for gastrointestinal stenosis due to corrosive agents [32]. Although the success rate for esophageal strictures may be initially high as to up 85% [33], following endoscopic dilations, the chance of relapse is high and repeated procedures are often required. Endoscopic dilations results are also good in the short-term period for gastric strictures, but the mid- and long-term therapeutic efficacy is often unsatisfactory [34]. The therapeutic success depends on both number and frequency of dilation, balloon pressure, and the anatomy of the strictures; although it has been suggested a step-up dilation approach carried out every 2 weeks [35], it must be taken into account the fact that dilations can increase the fibrotic activity of the second phase of wound healing. Thus, intralesional corticosteroid therapy has been added to dilations, showing beneficial effect for refractory strictures due corrosive agents [36–40].

Over the last decade endoscopic stent insertion has been proposed as an alternative treatment to repeated dilations for refractory benign esophageal stricture, prior to surgery.

Thus, the self-expandable plastic (SEPSs) and metallic (SEMSs) stents have been used successfully as treatment of benign strictures of the GI tract [14, 34, 41]. The use of SEPS presents some advantages over SEMS, such as the absence of a metal mesh and the completely covering silicone membrane, that reduce mucosal overgrowth at the stent edges and allow safe removal, even though the stent has been in place for few months. Furthermore SEPSs, by a wider surface and an higher expansive force, reduce the risk of dislocation [41–43]; the limit of SEPS is that it is possible to place them only into esophageal strictures. 

SEMSs are commonly used for palliative treatment of patients with malignant dysphagia, and have dramatically reduced the morbidity of esophageal stent insertion compared with that associated to the placement of rigid plastic stents [44]. Also, SEMSs have been successfully used for palliative treatment of malignant gastric outlet obstruction [45]. 

SEMSs have never been proposed for benign caustic strictures of pyloric region, as this kind of strictures is relatively uncommon and also because the covered (totally or partially) metal enteral stents have been available just recently. The choice between totally or partially covered stent is secondary to the stricture morphology and the probability of stent dislocation, depending on how much the tract is narrow and straight.

Here we report our experience with SEMS for the treatment of caustic upper GI strictures. We used SEMS since we have a good experience (109 metallic stents positioned in the last 5 years, with 12 of these procedures carried out in patients with benign GI tract diseases. In these patients SEMS were left in place for periods of 1–3 months, with good results) in temporary SEMS placement for treatment of esophageal, gastrojejunal anastomotic, and peptic pyloric strictures.

The main problem for temporary metallic stent is to determine the optimal placement period. Caustic strictures are usually difficult to treat because the granulation tissue is very strong and plentiful; therefore, the longer the stent is left in place, the more difficult is to remove it [46]. In our experience, we observed that, later than 4 weeks from stent placement, granulation tissue grows within the distal noncovered areas of the stent and within the stent covered trunk, as a consequence of the silicon cover usury. Moreover, we realized that it is impossible to remove stents without the use of APC in a procedure that is surely time consuming (about 60–90 minutes).

Endoscopy is the first therapeutic approach of caustic stenosis with either step-up mechanical or pneumatic dilations; *moreover, the placement of endoscopic stents, that may be subsequently removed, has been established as an effective way to treat benign refractory strictures due to corrosive agents* [47, 48]. In our cases SEMS positioning was effective, probably due to the stent radial expansive force exerted, and in selected cases, it might represent a new alternative treatment for patients with caustic esophagogastric strictures. Transient placement of a SEMS could significantly improve prognosis of patients with benign stenoses refractory to dilation procedure. By the use of APC, SEMS can be removed after a long period despite the development of tissue overgrowth at the edges of the stent. This procedure must be performed by skilled hands in referral endoscopic centers. Further studies, also with the new biodegradable stents, are needed in order to confirm the efficacy of this conservative endoscopic technique [49].

## Figures and Tables

**Table 1:** Zargar endoscopic classification for caustic mucosal injury (adapted from [7]).

Grade	Definition
0	normal findings at endoscopy
1	edema and hyperemia of mucosa
2A	friability, hemorrages, erosions, blisters, whitish membranes, exudates and superficial ulcerations
2B	grade 2A plus deep discrete or circumferential ulcerations
3A	small scattered multiple ulcerations and areas of necrosis (brown-black or greyish discoloration)
3B	extensive necrosis
